# Endothelial nitric oxide synthase (−786T>C) polymorphism and migraine susceptibility: A meta-analysis

**DOI:** 10.1097/MD.0000000000012241

**Published:** 2018-09-07

**Authors:** Han Dong, Zhi Hao Wang, Bin Dong, Ya Nan Hu, Hui Ying Zhao

**Affiliations:** a Department of Geriatric Medicine, The First Hospital of Jilin University, Changchun; b College of Basic Medical Science, Changchun University of Chinese Medicine, Jilin Province, China.

**Keywords:** endothelial nitric oxide synthase, migraine, polymorphism, susceptibility

## Abstract

**Background:**

The aim of this study was to evaluate the correlation between endothelial nitric oxide synthase (eNOS) polymorphism (−786T>C) and migraine susceptibility in a meta-analysis.

**Methods:**

A literature search was performed for case–control studies from inception to July 30, 2018 focusing on eNOS polymorphism (−786T>C) and risk of migraine. From 454 full-text articles, 6 were included in this study. Heterogeneity was assessed with the *I*^2^ index and quality assessment was performed using the Newcastle–Ottawa scale.

**Results:**

CC genotype was not related to higher susceptibility of migraine compared with TT+ TC genotypes with significant difference (fixed effects model; OR = 1.27; 95% CI = 0.90–1.80; *P = *.17; *I*^2^ = 18%). However, subgroup analysis showed CC variant increase the risk for migraine compared with TT+ TC genotypes in Caucasian populations (fixed effects model; OR = 1.62; 95% CI = 1.03–2.56; *P = *.04; *I*^2^ = 18%), which could not be observed in non-Caucasian populations (fixed effects model; OR = 0.88; 95% CI = 0.51–1.53; *P = *.66; *I*^2^ = 0%). There was no significant difference for other genotypes and alleles between patients with migraine and healthy controls (all *P > *.05).

**Conclusion:**

This meta-analysis indicated that CC variant increases the risk for migraine compared with TT + TC genotypes in Caucasian populations.

## 1 Introduction

Migraine is a chronic paroxysmal neurological disorder characterized by attacks of moderate or severe headache and reversible neurological and systemic symptoms that affects approximately 12% to 16% of the population.^[1,2]^ Migraine often cause significant disability and impaired quality of life, adversely affecting the activities of daily living and work-related productivity in many patients.^[3]^ Migraine is clinically diagnosed according to the criteria set out by the International Headache Society (IHS).^[4]^ Migraine often begins with premonitory symptoms hours or days before the onset of pain. The most common premonitory symptoms include fatigue, impaired concentration, and neck stiffness. Furthermore, other psychological (anxiety, depression, irritability), arousal (drowsiness), neurological (photophobia), and cranial parasympathetic symptoms (lacrimation), and general symptoms (e.g., yawning, increased urination, nausea, diarrhoea) can occur before the onset of pain.^[2]^ The IHS classifies migraine as migraine with aura (MA), representing about one-third of all migraine, or migraine without aura (MO) that accounts for approximately two-thirds of all migraine in the population.^[2,4,5]^ Although researches have been performed for many years, the complex pathogenetic mechanisms of migraine still remain unclear. Migraine is considered to be as a result of combination of environmental and genetic aspects.

Nitric oxide (NO) is a signaling molecule with a short half-life, which is synthesized from L-arginine by 3 isozymes of nitric oxide synthase (NOS), including neuronal NOS (nNOS), endothelial NOS (eNOS), and inducible NOS (iNOS).^[6]^ It exerts a variety of physiological effects such as regulating blood pressure via smooth muscle relaxation, and functioning as a neurotransmitter.^[7]^ NO in the migraine pathogenesis has been reported in several studies.^[8,9]^ Polymorphism of NOS could affect production of NO, which may be connected with migraine. The associations between a single nucleotide polymorphism (SNP) of NOS and migraine were widely studied.^[10–20]^ Although nNOS is predominately expressed in neurons of the central and peripheral nervous systems, studies evaluating the association between nNOS polymorphism and migraine were limited. There was no marked association between nNOS polymorphisms (276C>T, rs2682826; −421+145599C>T, rs693534; 853–2109C>A, rs7977109) and migraine susceptibility.^[10,11]^ Conversely, a series of studies reporting the relationships between SNPs of iNOS, eNOS genes, and risk of migraine.^[12–20]^ There was no association between bi-allelic tetranucleotide polymorphism of iNOS and migraine.^[12]^ The 2087G>A (rs2297518) and the −1026C>A (rs2779249) polymorphisms in the iNOS gene affect the susceptibility to MA when their effects are combined within haplotypes, whereas the 2087G>A affects the susceptibility to aura in migraine patients.^[13]^ A significant interaction between iNOS 2087G>A and eNOS 2512+242G>A/C (rs743506) polymorphisms in migraine patients compared to control group, suggesting that this combination affect migraine susceptibility.^[14]^ Three clinically relevant polymorphisms of eNOS have been studied: SNP in the promoter region (−786T>C, rs2070744), an SNP in exon 7 (+894G>T, rs1799983), and the variable number of tandem repeats (VNTR) in intron 4.^[21,22]^ Borroni et al^[15]^ reported that eNOS (+894G>T) polymorphism was an independent risk for MA. However, a later study could not verify the conclusion.^[16]^ A recent meta-analysis indicated that “T” allele of the eNOS +894G > T variant increased the risk of migraine among non-Caucasians.^[17]^ The substitution of thymine by cytosine in the 5′ promoter region at nucleotide—786 (−786T>C) results in a significant reduction in eNOS gene promoter activity and in decreased basal NO production.^[16]^ Similarly, there are some controversies for the connection between eNOS polymorphism (−786T>C) and migraine risk.^[14,18–20]^ This meta-analysis was carried out to evaluate the relationship between eNOS (−786T>C) polymorphism and migraine susceptibility.

## 2 Patients and methods

### 2.1 Literature search

Two authors independently performed literature search for published studies from inception to July 30, 2018 using PubMed, Cochrane Database, EMBASE, Web of science, Google scholar, and China National Knowledge Infrastructure (CNKI). The keywords used were “nitric oxide synthase,” or “NOS,” or “endothelial nitric oxide synthase,” or “NOS3,” or “eNOS” and “migraine,” or “migraine disorder.” The titles and abstracts of the resulting articles were examined and unrelated articles were excluded. If an article was selected for inclusion, the references were reviewed for additional studies. This study was approved by the ethics committee of the First Hospital of Jilin University.

### 2.2 Inclusion and exclusion criteria

An article was relevant if it reported original data from case–control study, regardless of language, investigating the correlations between eNOS polymorphism (−786T>C) and migraine susceptibility. Controls were healthy individuals without migraine randomly selected from general populations after excluding those with underlying diseases (e.g., cardiovascular, renal, hepatic, gastrointestinal, pulmonary, endocrine, auto-immune, psychiatric diseases),^[1,5,14,19]^ or whose health was established by medical diagnosis.^[18,20]^ Studies were excluded if one of the following existed: studies with insufficient genotyping data of patients or control group; case reports; review articles; experiment researches; case-only studies. Disagreements between the 2 reviewers regarding inclusion of a study were resolved through consensus.

### 2.3 Data extraction and quality assessment

Two independent reviewers extracted the data and assessed the quality of each study using the Newcastle–Ottawa scale (NOS). In this scale 9 points represent the highest quality. In case of disagreements, the original article was evaluated by a third reviewer.

### 2.4 Data analysis

RevMan 5.2 (Cochrane database) was used to analyze the data. The results were reported as odds ratios (OR) with 95% confidence intervals (CI). Heterogeneity between studies was assessed by using the *I*^2^ statistic: values of 25%, 50%, and 75% represent mild, moderate, and severe heterogeneity, respectively. Based on results of the heterogeneity test, a fixed effect model was used if *P > *.10, while a random effects model was performed if *P* ≤ 0.10. Begg's funnel test and Egger's test were applied to evaluate publication bias across studies with Stata (version 12.0). A *P < *.05 was considered statistically significant.

## 3 Results

### 3.1 Search results

**Figure 1:**
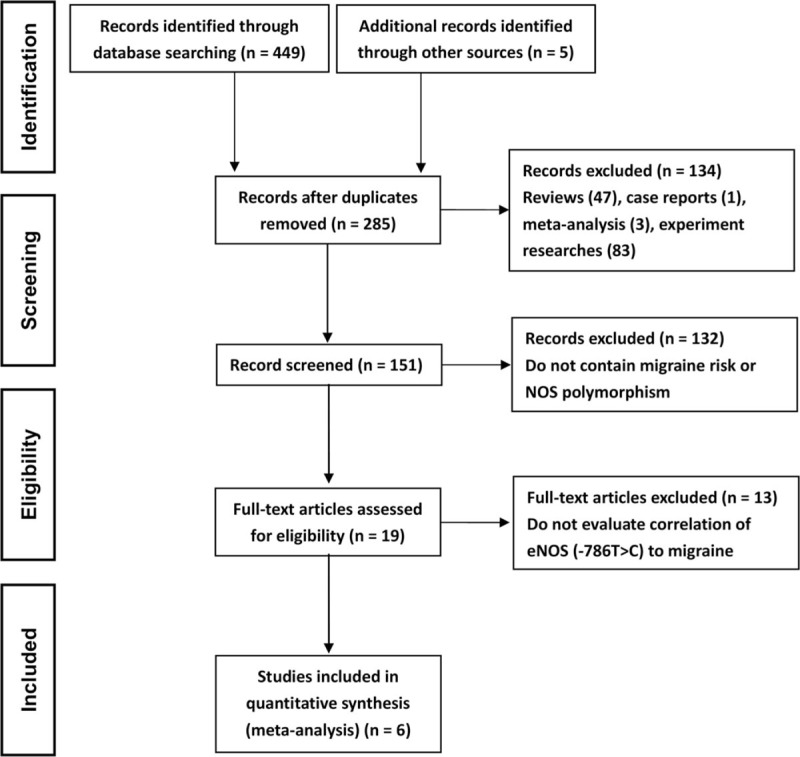
Flow chart of literature selection in this study.

We followed the PRISMA (Preferred Reporting Items for Systematic Reviews and Meta-Analyses) statement for reporting our results. Six studies of full-text articles were selected for inclusion in this meta-analysis. Our selection process for studies included in the analysis was shown in Figure 1.

### 3.2 Characteristics of the included studies

The characteristics of the included studies, which were published between 2005 and 2017, were presented in Table 1. Two studies were from Brazil,^[14,19]^ 2 from Turkey,^[1,5]^ 1 from the United Kingdom,^[18]^ and the last one was from Iran.^[20]^ A total of 763 patients with migraine and 560 healthy controls were included in this meta-analysis. The frequencies of genotypes and alleles were summarized in Table 2. All studies had a Newcastle–Ottawa scale score ≥7, with an average of 7.5 (Table 1).

**Table 1:**
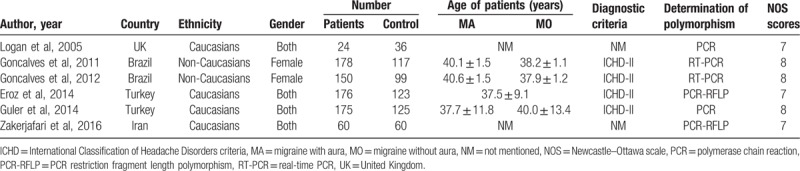
Characteristics of the included studies.

**Table 2:**
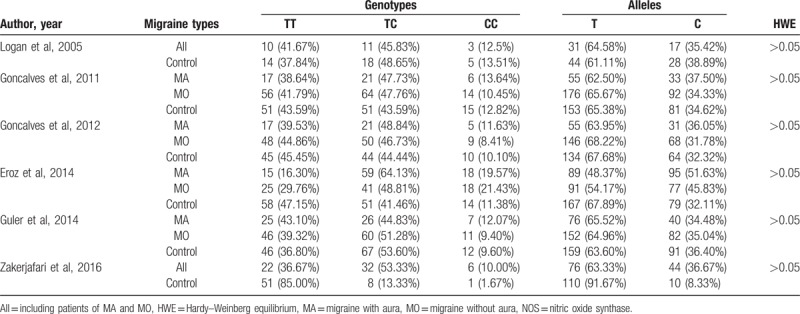
The detailed frequencies of genotypes and alleles of NOS.

### 3.3 Correlation of eNOS polymorphism −786T>C and migraine susceptibility

**Figure 2:**
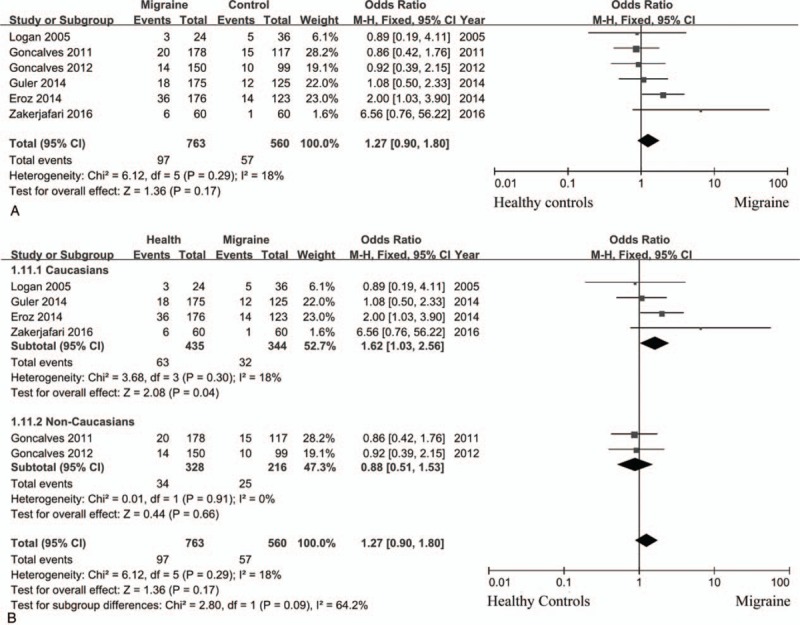
eNOS (−786T>C) polymorphism and the risk of migraine (CC vs TT + TC). (A) the overall results; (B) different populations.

**Figure 3:**
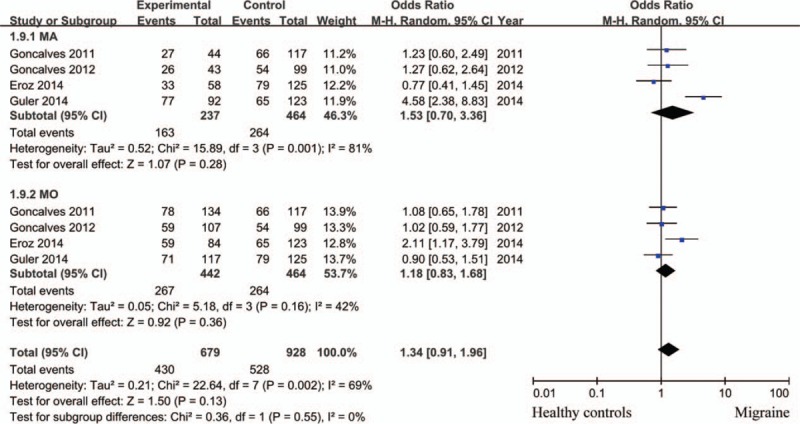
eNOS (−786T>C) polymorphism and the risk of migraine subtypes (TT vs TC + CC).

**Figure 4:**
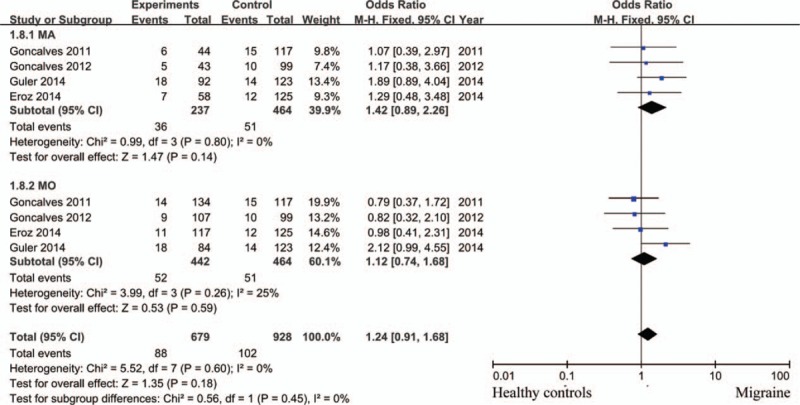
eNOS (−786T>C) polymorphism and the risk of migraine subtypes (CC vs TC + TT).

Compared with patients with TT genotype, those with CC + TC genotypes did not have higher risk for incidence of migraine with statistical significance (random effects model; OR = 1.66; 95% CI = 0.88–3.13; *P = *.12; *I*^2^ = 86%). CC genotype was not associated with higher susceptibility of migraine compared with TT+ TC genotypes with significant difference (fixed effects model; OR = 1.27; 95% CI = 0.90–1.80; *P = *.17; *I*^2^ = 18%) (Fig. 2A). Similar result was observed when analyzed patients with C allele and those with T allele (random effects model; OR = 1.30; 95% CI = 0.91–1.85; *P = *.15; *I*^2^ = 76%). However, subgroup analysis showed CC variant increase the risk for migraine compared with TT+ TC genotypes in Caucasian populations (fixed effects model; OR = 1.62; 95% CI = 1.03–2.56; *P = *.04; *I*^2^ = 18%), which could not be observed in non-Caucasian patients (fixed effects model; OR = 0.88; 95% CI = 0.51–1.53; *P = *.66; *I*^2^ = 0%) (Fig. 2B). We also analyzed the relationship of eNOS polymorphism and migraine subtypes, MA and MO, no significant difference was observed (Fig. 3: TT vs TC + CC; Fig. 4: CC vs TC + TT).

### 3.4 Heterogeneity

**Figure 5:**
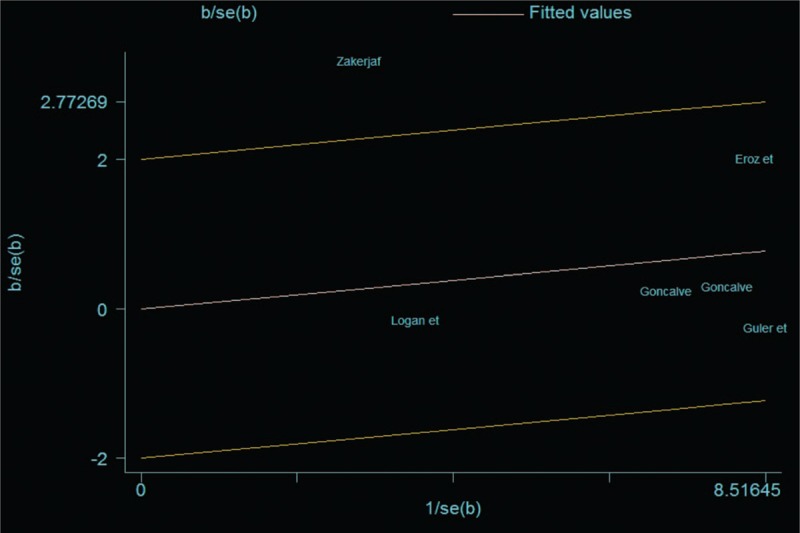
Galbraith plots for studies included in this meta-analysis.

Considering heterogeneity existed in this study, we used Galbraith plots to explore the heterogeneity sources (Fig. 5). Low Newcastle–Ottawa scale score^[20]^ and different genetic backgrounds^[5]^ were the main causes for heterogeneity. After removal of these 2 studies, as concluded above, both CC + CT genotypes (fixed effects model; OR = 0.99; 95% CI = 0.76–1.30; *P = *.95; *I*^2^ = 0%) and C allele (fixed effects model; OR = 0.98; 95% CI = 0.81–1.20; *P = *.86; *I*^2^ = 0%) did not increase the risk of migraine compared with TT genotype and T allele, respectively.

### 3.5 Publication bias

**Figure 6:**
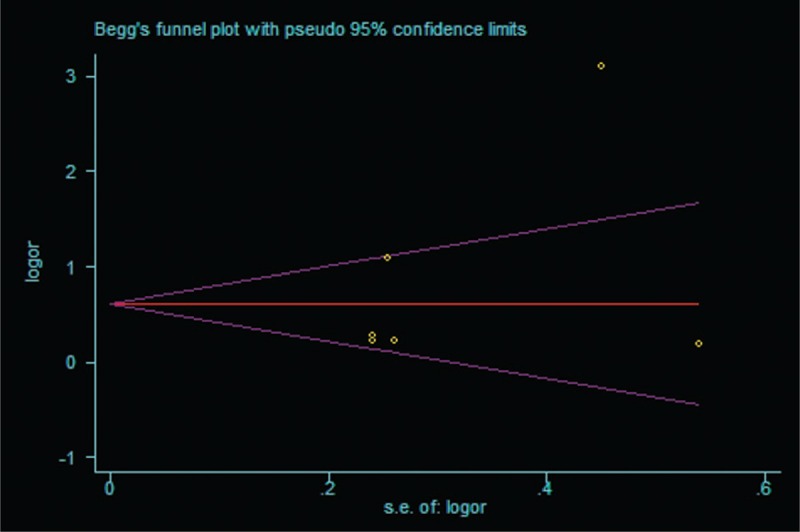
Begg's funnel plot for publication bias.

Publication bias was determined using Begg's funnel plots and Egger's test. Neither Egger's test nor Begg's funnel plots indicated any significant publication bias (*P = *.452 and 0.317, respectively) (Fig. 6).

## 4 Discussion

NO regulates a host of physiological functions, including vascular tone, pain sensation, neurotransmission, and as an immune defence mechanism.^[23]^ NO activates soluble guanylate cyclase and subsequently increases cGMP. cGMP can induce gene expression via the induction of transcription factors such as mitogen activated protein (MAP) kinases or NF-κB.^[24]^ Although the aetiology and pathogenesis of migraine remain unclear, NO is believed to play a key role in migraine pathogenesis. Migraine was initiated by NO donors, glyceryl trinitrate (GTN), or isosorbide dinitrate, which indicated that NO may participate in the pathological process of migraine.^[24,25]^ GTN did initiate migraine headaches but not the aura in migraineurs with aura in most studies. This indicated that NO is involved in the pain mechanism in both types of migraines but not in the initiation of the aura.^[24]^ Further study demonstrated that not NO itself but the elevation of cGMP by NO or other mediators is necessary to induce migraine.^[26]^ Inhibition of NOS in spontaneous migraine attacks leads to the attenuation of symptoms in two-thirds of the patients.^[27]^ Thus, NO seems not only be involved in the initiation of migraine attacks but also in the maintenance of these attacks. Calcitonin gene related peptide (CGRP) is a key player in migraine pathophysiology.^[28]^ CGRP levels were elevated following GTN-induced migraine.^[29]^ Pretreatment of trigeminal ganglia cultures with NO donors was also found to increase CGRP release in response to both depolarization and inflammatory mediators, which may have occurred due to NO donors increasing CGRP promoter activity.^[30,31]^ While NO donors have some functions independent of CGRP, their function in migraine may strongly relate to promoting CGRP production and release, adding to the CGRP-influenced pathways in migraine.^[31]^

NO was synthesized by NOS from L-arginine. The activity of NOS has an important impact on the production of NO, and polymorphism of NOS gene leads to variant activity of NOS. In this study, we carried out a meta-analysis to evaluate the correlation of eNOS polymorphism (−786T>C) and migraine risk. The overall results did not indicate associations between eNOS polymorphism and migraine. However, subgroup analysis showed CC variant increase the risk for migraine compared with TT + TC genotypes in Caucasian populations.

When compared between CC + TC genotypes and TT genotype, between T allele and C allele, significant heterogeneity existed. Random effects model indicated that there was no significant difference between patients and healthy controls. First, we carried out subgroup analysis by populations. Lack of association was observed between eNOS polymorphism and migraine susceptibility in non-Caucasians (fixed effects model; OR = 1.10; 95% CI = 0.78–1.56; *P = *.58; *I*^2^ = 0%). Second, using Galbraith plots, source of heterogeneity was identified. After removal of studies with significant heterogeneity, fixed model showed that both CC + CT genotypes and C allele did not increase migraine susceptibility compared with TT genotype and T allele, respectively.

For migraine subtypes, 4 studies were included.^[1,5,14,19]^ We tried to contact the authors of the remaining 2 studies for details. However, no reply was received. Strategies mentioned above were used to cope with study of high heterogeneity. Again no relationship was observed between eNOS gene polymorphism and the risk of migraine subtypes regardless of subgroup analysis, fixed or random effects model usage.

Our study has several strengths. First, this is the largest study to date evaluating the correlation between eNOS −786T>C polymorphism and risk of migraine. Second, patients and controls of different genetic backgrounds made it possible to analyze the associations in different populations. However, our study also has several limitations. First, due to apparent heterogeneity across studies in some cases, the findings from our study should be dealt with some caution. Second, we did not include academic dissertations and conference papers, so there may have been bias in provision of data. Third, there are other factors that affect the incidence of migraine, for example, gender,^[32]^ medication overuse,^[33]^ and so on. However, we could not evaluate the influence of these factors on the association between eNOS polymorphism and migraine susceptibility with studies included in this meta-analysis. Furthermore, a tendency of higher risk for migraine susceptibility could be observed in this meta-analysis (e.g., CC genotype vs TC + TT genotypes, C allele vs T allele). Although statistical significance could not be acquired with current studies, a larger sample may reach a positive result. Hence, further study is still needed.

Although the association of eNOS −786T>C polymorphism and migraine susceptibility were reported in previous studies. This meta-analysis indicated that CC variant increase the risk for migraine compared with TT+ TC genotypes in Caucasian populations. More studies are still needed to verify such conclusion.

## Acknowledgments

We thank the Department of Geriatric Medicine, the First Hospital of Jilin University, for their assistance in this work. This work was supported by the Program from Health commission of Jilin Province (2014Q030).

## Author contributions

DH, WZH, DB, HYN, and ZHY conceived the study. DH, WZH, DB, HYN, and ZHY designed the study and analyzed the data. DH, DB, and ZHY wrote this manuscript. All authors discussed and revised the manuscript before submission.

**Conceptualization:** Han Dong.

**Data curation:** Han Dong, Bin Dong, Ya Nan Hu, Hui Ying Zhao.

**Formal analysis:** Han Dong, Zhi Hao Wang, Bin Dong.

**Funding acquisition:** Han Dong.

**Investigation:** Han Dong, Bin Dong, Ya Nan Hu, Hui Ying Zhao.

**Methodology:** Han Dong, Zhi Hao Wang, Bin Dong, Ya Nan Hu, Hui Ying Zhao.

**Resources:** Zhi Hao Wang.

**Software:** Han Dong, Zhi Hao Wang, Bin Dong.

**Supervision:** Hui Ying Zhao.

**Validation:** Ya Nan Hu.

**Writing – original draft:** Han Dong.

**Writing – review & editing:** Han Dong.
